# Molecular characterization of feline astrovirus in domestic cats from Northeast China

**DOI:** 10.1371/journal.pone.0205441

**Published:** 2018-10-09

**Authors:** Shushuai Yi, Jiangting Niu, Hualei Wang, Guoying Dong, Yanbing Guo, Hao Dong, Kai Wang, Guixue Hu

**Affiliations:** 1 College of Animal Science and Technology, Jilin Agricultural University, Changchun, Jilin Province, China; 2 Key Laboratory of Jilin Province for Zoonosis Prevention and Control, Military Veterinary Research Institute, Academy of Military Medical Sciences, Changchun, Jilin Province, China; 3 College of Global Change and Earth System Science, Beijing Normal University, Haidian, Beijing; 4 Jilin Institute of Animal Husbandry and Veterinary Science, Changchun, Jilin Province, China; 5 College of Life Science and Technology, Jilin Agricultural University, Changchun, Jilin Province, China; Nanjing Agricultural University, CHINA

## Abstract

Feline astrovirus (FeAstV) which belonged to the genus *Mamastrovirus* was first identified in the feces of kittens with diarrhea in the USA in 1981 by electron microscopy, and had been reported in many countries. Presently, there are no any reports of the circulation of FeAstV in mainland China. We performed this study to investigate the apparent prevalence and genetic variability of FeAstV infected in cats in mainland China for the first time. We tested fecal samples of 105 cats with diarrhea and 92 asymptomatic cats in five cities in northeast China by RT-PCR targeting RNA-dependent RNA polymerase (*RdRp*) gene of FeAstV, and analyzed sequences variability and phylogenetic evolution based on the complete capsid gene of FeAstV strains obtained from positive samples. The overall prevalence of FeAstV was 23.4% (46/197) of which 38 were tested in cats with diarrhea (36.2%, 38/105) and 8 were in asymptomatic cats (8.7%, 8/92). Mixed infection with other enteroviruses including feline parvovirus (FPV), feline bocavirus (FBoV) and feline kobuvirus (FeKoV) was found in 38 FeAstV-positive samples. Phylogenetic analysis based on the complete capsid gene revealed all FeAstV strains were divided into two different groups with a 0.454±0.016 of mean amino acid genetic distance between two groups, suggesting that FeAstVs should be classified into two different genotype species. This study provided the first molecular evidence that FeAstV with considerable genetic diversity was circulating in northeast China, and analyzed genetic variability and classification of FeAstVs for the first time.

## Introduction

Astroviruses (AstVs), which are taxonomically classified within the family *Astroviridae*, are small, non-enveloped, spherical virus of about 28–30 nm in diameter [1]. AstV has a positive single-stranded RNA genome of about 6.8–7.3 kb in length that contains three overlapping open reading frames (ORFs), designated ORF1a, ORF1b and ORF2, and a poly A tail. ORF1a and ORF1b are located at the 5’ end of the genome and together encode the non-structural proteins which include a 3C-type serine protease, a viral genome-linked protein (*VPg*), RNA-dependent RNA polymerase (*RdRp*) and several uncharacterized proteins [2]. At the 3’-terminal end, ORF2 encodes the astrovirus capsid protein [3]. The family *Astroviridae* is officially divided into two genera, *Mamastrovirus* and *Avastrovirus*, which infect mammals and avian species, respectively. According to the recent report of the International Committee on Taxonomy of Viruses (ICTV), 19 different viral species are recognized within the genus *Mamastrovirus*, as well as 3 species within *Avastrovirus* [1]. However, members of the family *Astroviridae* are constantly updated with the discovery of novel viruses from other animal species and the different genotypes. To date, 33 and 7 distinct genotype species of *Mamastrovirus* and *Avastrovirus*, respectively, have been proposed [4].

AstVs are known as one of the important pathogens associated with either mild or severe gastroenteritis in human, especially in young children and immunodeficient individuals [5,6]. However, it is unclear whether AstVs infections are associated with enteric disease in other mammals, because they were not only detected in animals with enteric disease, but also found in healthy animals, including dogs, piglets, cats and others [7–9]. Recently, extraintestinal diseases that caused by AstVs have been reported in many mammals, such as the encephalitis and neurological disorders in human and cattle, shaking syndrome in mink, as well as congenital tremor and respiratory disease in piglets [10–13].

Feline astrovirus (FeAstV), a officially recognized species (namely mamastrovirus 2) in the genus *Mamastrovirus*, was first reported in 1981 by electron microscopy in fecal samples from domestic cats with diarrhea in USA [14], and the first molecular characterization of FeAstVs was reported in 1998 [15]. To date, FeAstVs have been identified in domestic cats, with or without diarrhea, in the USA [14], Britain [15], Australia [16], Germany [17], Hong Kong [18] and south Korea [9]. However, there are no any data about FeAstVs infection in cats in mainland China, as well as the genotypes of FeAstVs circulated worldwide are still unclear. The aim of this study was to investigate the prevalence and genetic diversity of FeAstVs in domestic cats in Northeast China, and the relationship between enteric disease in cats and FeAstV infection.

## Materials and methods

### Fecal samples

Between January 2016 and November 2017, fresh fecal samples were collected from 197 domestic cats of which 105 were diarrheal and 92 were asymptomatic from private veterinary clinic and animal shelter centre in five different cities (Shenyang, Jinzhou, Changchun, Jilin and Harbin) of northeast China. Individual fresh feces were immediately placed in RNase-free tubes and were stored at -70°C until further use. The study was reviewed and approved by the Animal Care and Use Committee of Jilin Agricultural University.

### Genome extraction

Fresh feline feces were homogenized in phosphate buffered-saline solution (PBS) at a concentration of about 0.5 g/ml, and then the supernatant was collected after centrifugation (10,000 g for 10 min). Sample’s genome was extracted using AxyPrep body fluid viral DNA/RNA miniprep kit (AXYGEN, China), and then reverse transcribed to synthesize cDNA using the RevertAid first strand cDNA synthesis kit (Invitrogen, USA) according to the manufacturer’s instructions. The synthesize cDNA was stored at -70°C until further testing.

### Detection of FeAstV and other feline enteroviruses

The RT-PCR was performed using a pair of specific primers, FeAstVAF and FeAstVAR, targeted 418 bp of *RdRp* gene in ORF1b for FeAstV in a thermo cycler (BIOER, China) to detect FeAstV. Amplified conditions were set as previously described [19]. Then, FeAstV-positive samples were examined for feline bocavirus (FBoV), feline parvovirus (FPV) and feline kobuvirus (FeKoV) using PCR/RT-PCR assays previously described [19,20]. The apparent prevalence of FeKoV was calculated, and the association with clinical symptoms and regions were also analyzed by Chi Square test using the statistical program PASW Statistics 19.0. Furthermore, PCR products of FeAstV-positive samples were purified using AxyPrep DNA gel Extraction kit (CORNING, China) according to the manufacturer’s instruction, and then were sent to Sangon Biotech (Shanghai, China) for sequencing.

### FeAstV ORF2 gene amplification and sequencing

In order to further analyze the genetic characteristics of FeAstVs circulated in northeast China, the complete ORF2 gene was amplified using a primer set (forward: 5’- ATGGCTAGCAAGYCTGGYAAAGAAG-3’; reverse: 5’-GCGTGGCCTCGGCTCTCAA-3’) that we designed and optimized in the current study. The amplification was performed in a thermo cycler using the following conditions: 94°C for 5 min; 35 cycles of 94°C for 1 min, 65°C for 1 min and 72°C for 2 min; and a final extension at 72°C for 10 min. PCR products were analyzed on 1.0% agarose gels, and the positive fragment with size of 2438 bp was purified using AxyPrep DNA gel Extraction Kit (AXYGEN, China), and then cloned into a PMD-18T vector (TAKARA, China). At least three positive clones for each sample were sent to Sangon Biotech (Shanghai, China) for sequencing.

### Phylogenetic analyses

The nucleotide and deduced amino acid sequences of partial *RdRp* gene and full-length ORF2 gene obtained in this study were aligned using the ClustalW methods in MEGA 7.0 software, and phylogenetic trees were constructed using the neighbor-joining method with 1,000 bootstrap replicates. Variability analysis based on amino acid sequence of the complete ORF2 gene was performed using online Protein Variability Server software (http://imed.med.ucm.es/PVS/). The amino acid distance of FeAstVs was estimated using the p-distance model with 1,000 bootstrap replicates in MEGA 7.0 software.

## Results

### Apparent prevalence of FeAstV in fecal samples

We examined a total of 197 fecal samples collected from five different cities in northeast China of which 46 (23.4%) were tested to be positive for FeAstV (Table 1). The apparent prevalence of FeAstV in cats from different cities was ranged from 17.6% to 28.2%, and the difference was not statistically significant (*p* = 0.721). Cats from private veterinary clinics (21.1%, 15/71) and from animal shelter centre (24.6%, 31/126) had a similar FeAstV prevalence. The apparent prevalence of FeAstV tested in cats with and without diarrhea was 36.2% (38/105) and 8.7% (8/92), respectively, and the apparent prevalence of FeAstV infection in cats with diarrhea was significantly higher than that in asymptomatic cats (Chi Square test, χ^2^ = 20.711, *p*<0.001). Detail information of FeAstV-positive samples identified in this study was shown in S1 Table. Moreover, we tested other feline enteroviruses including FBoV, FPV and FeKoV in these FeAstV-positive samples, and screening results were shown in Table 2. Among the 46 FeAstV-positive samples, 38 were co-infected with other feline enteroviruses while 8 were infected with FeAstV alone. In FeAstV-positive samples from cats with diarrhea, 86.8% (33/38) were co-infected with other feline enteroviruses of which 28 were co-infected with FPV. While in FeAstV-positive samples from asymptomatic cats, 62.5% (5/8) were tested to be positive for FBoV and/or FeKoV, but no was positive for FPV.

**Table 1:** The apparent prevalence of feline astrovirus infection in cats in this study.

Factor		Region					Source		Clinical symptoms		Total
Category		Shenyang	Jinzhou	Changchun	Jilin	Harbin	PVC	ASC	Diarrhea	Normal	
**Number of tested samples**		36	17	85	33	26	71	126	105	92	197
**Number of positive samples**		7	3	24	7	5	15	31	38	8	46
**Prevalence**		19.4%	17.6%	28.2%	21.2%	19.2%	21.1%	24.6%	36.2%	8.7%	23.4%
**Chi Square test**	**χ**^**2**^	2.080					0.307		20.711		
	***p***	0.721					0.580		<0.001		

PVC, private veterinary clinics; ASC, animal shelter centre.

**Table 2:** Screening results of other feline enteroviruses in FeAstV-positive samples in this study.

	Number of FeAstV positive samples	The number of co-infection in FeAstV-positive samples							
		None	FPV	FBoV	FeKoV	FPV+ FBoV	FPV+ FeKoV	FBoV+FeKoV	FPV+FBoV+FeKoV
**Diarrheal cats**	38	5	13	3	1	7	4	1	4
**Asymptomatic cats**	8	3	0	3	2	0	0	0	0
**Total**	46	8	13	6	3	7	4	1	4

FeAstV, feline astrovirus; FPV, feline parvovirus; FBoV, feline bocavirus; FeKoV, feline kobuvirus.

### Phylogenetic analysis of partial *RdRp* gene

**Fig 1:**
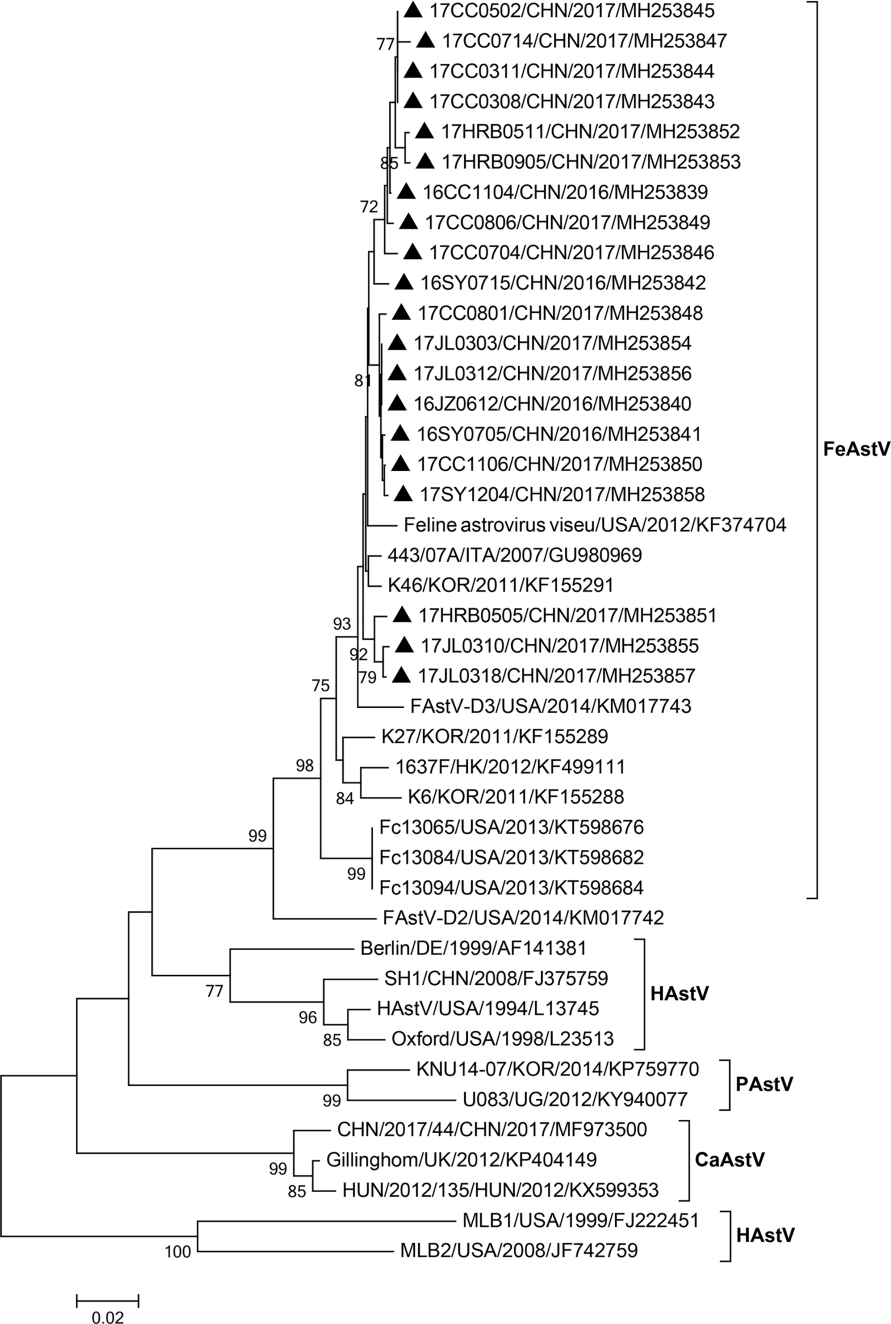
Phylogenetic tree of nucleotide sequences (336 bp fragment) from the RNA-dependent RNA polymerase (*RdRp*) gene of 20 FeAstV strains identified in this study (▲) and 11 FeAstVs and 11 other mamastroviruses reference sequences obtained from GenBank. The tree was constructed using the neighbor-joining method with 1,000 bootstrap replicates in MEGA 7.0 software.

Twenty FeAstV-positive samples were randomly selected and sequenced for partial *RdRp* gene (418 bp in 3’-terminal of ORF1b gene), and the sequences had been deposited in GenBank under accession numbers MH253839-MH253858. The nucleotide and deduced amino acid sequences of partial *RdRp* gene among the 20 FeAstV sequences and other astrovirus reference sequences were aligned using ClustalW 2. The 20 FeAstV sequences shared 94.3%-100% nucleotide identities and 96.4%-100% amino acid identities with each other, and displayed higher identities to FeAstV reference sequences obtained from GenBank at the nucleotide (91.1%-97.9%) and deduced amino acid (92.9%-100%) levels. Furthermore, the amino acid sequences of the FeAstV strains identified in this study shared 54.5%-81.2%, 69.6%-85.7% and 65.3%-88.7% identities with human astrovirus (HAstV), canine astrovirus (CaAstV) and porcine astrovirus (PAstV) reference strains, respectively. A neighbor-joining tree based on partial *RdRp* nucleotide sequences showed that all sequences identified in this study clustered together and belonged to FeAstV (Fig 1).

### Phylogenetic and variability characterization of the complete FeAstV capsid sequences

**Fig 2:**
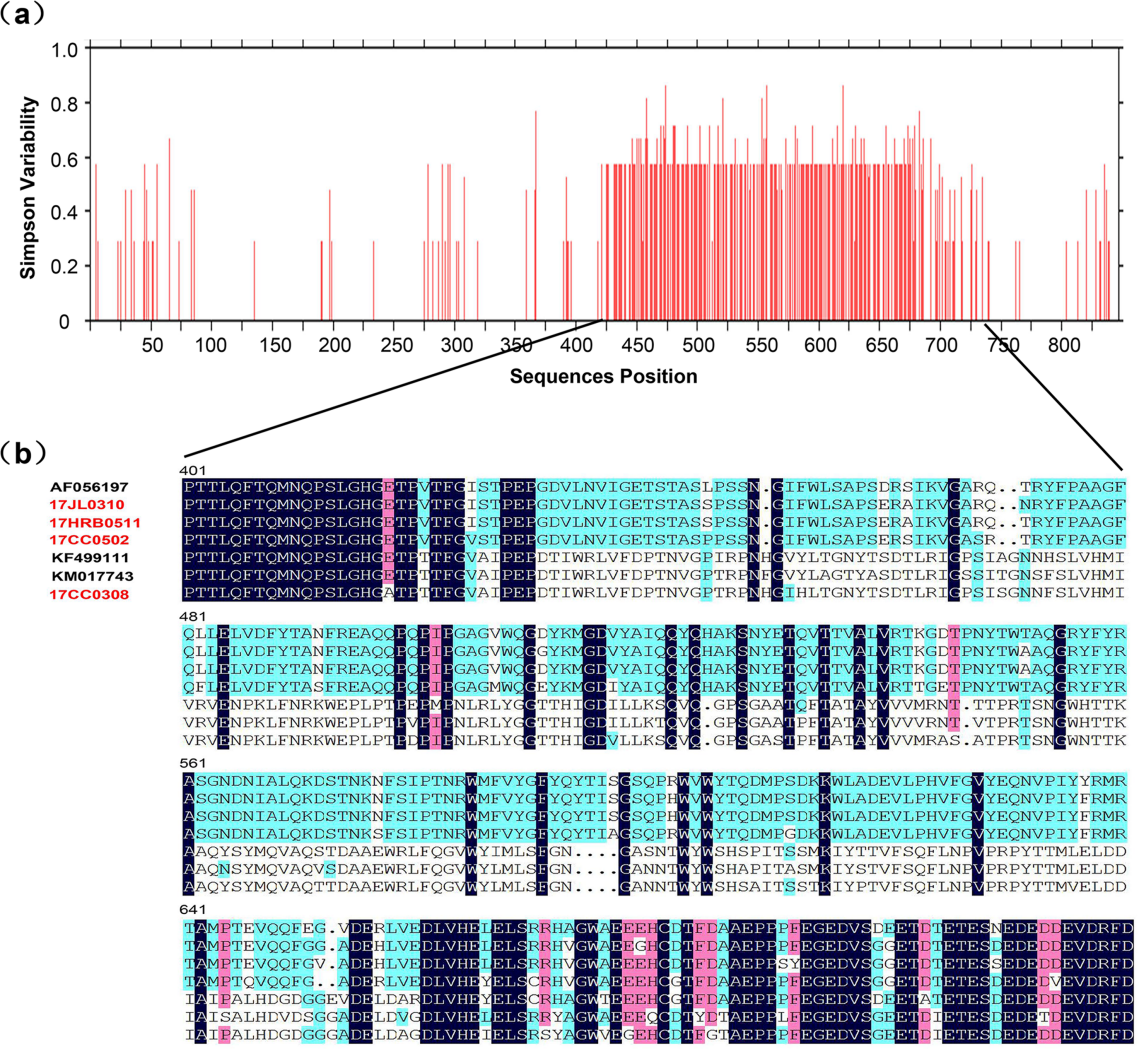
Variability analyses of capsid amino acid sequences among 20 FeAstV strains identified in this study and 5 FeAstV reference strains obtained from GenBank. (a) A variability scan for FeAstV capsid amino acid sequences was constructed using the Simpson diversity index in an online Protein Variability Server software (http://imed.med.ucm.es/PVS/). (b) Alignment of partial FeAstV capsid amino acid sequences (central region, from residues 401 to 720) between 4 representative strains identified in this study (red font) and 3 FeAstV reference strains.

**Fig 3:**
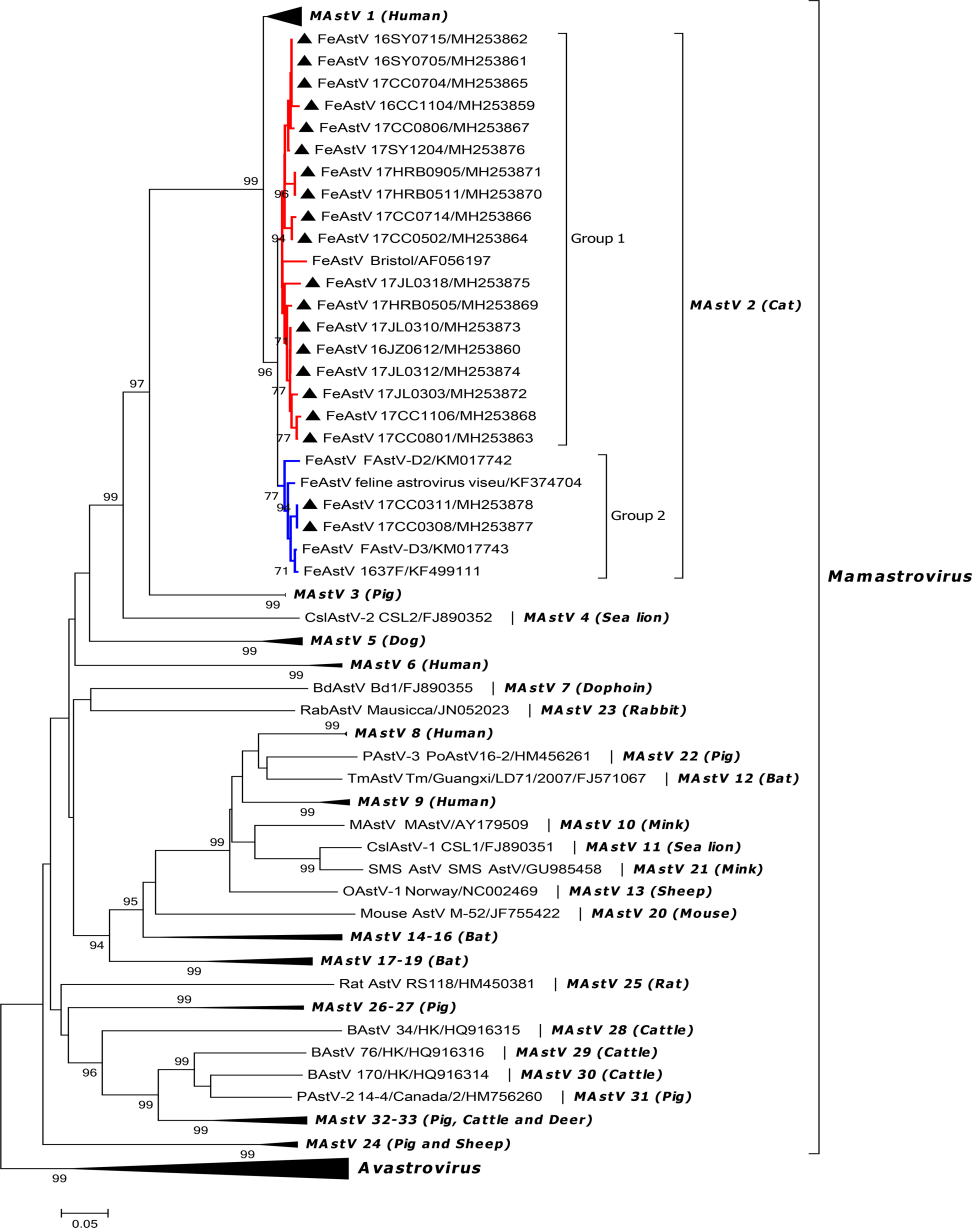
Phylogenetic analysis based on deduced amino acid sequences of the complete astrovirus ORF2 gene. The tree was generated using the neighbor-joining method in MEGA 7.0 software with 1,000 bootstrap values, and only bootstrap values >70% were displayed above the tree branches. Triangle indicates FeAstV strains identified in the present study. All FeAstV strains were divided into two groups, group 1 with red line and group 2 with blue line. Names in italics represent genotype species. Host(s) of origin is indicated in brackets.

To further describe the genetic characters of FeAstVs circulated in northeast China, the complete capsid gene (ORF2 gene) of the 20 samples which were used for sequencing and analyses of RdRp gene, was amplified and sequenced. The 20 sequences were successfully sequenced and were divided into three different types according to the length of sequences: i) 17CC0308 and 17CC0311 (GenBank accession numbers MH253877 and MH253878), the ORF2 gene was 2,445 nt in length and encoded the capsid protein of 814 aa; ii) 17CC0502 and 17CC0714 (GenBank accession numbers MH253864 and MH253866), the ORF2 gene was 2,448 nt in length and encoded the capsid protein of 815 aa; iii) other 16 FeAstV strains (GenBank accession numbers MH253859-MH253863, MH253865 and MH253867-MH253876), the ORF2 gene was 2,451 nt long and encoded the capsid protein of 816 aa. Pairwise nucleotide and deduced amino acid comparison showed that the nucleotide and amino acid identities among these strains were 76.8%-99.7% and 72.2%-99.4%, and these strains were 76.2%-89.0% and 71.1%-94.0% similar to other FeAstV reference strains at the nucleotide and deduced amino acid levels, respectively. Among these FeAstV strains identified in the present study, 17CC0308 and 17CC0311 shared more than 90% amino acid identities with FeAstV reference strains FAstV-D2 (KM017742), FAstV-D3 (KM017743), 1637F (KF499111) and feline astrovirus viseu (KF374704) while shared lower than 75% aa identities with reference strain Bristol (AF056197) identified in UK in 1998. Conversely, other 18 FeAstV strains identified in this study displayed >90% aa identities to reference strain Bristol, but <75% aa identities to other reference strains (S2 Table). Moreover, the complete capsid amino acid sequences of these FeAstV strains shared 55.4%-65.2%, 50.3%-51.3%, 38.4%-40.0% and 35.4%-37.4% identities with reference strains in the species MAstV 1 and MAstV 3–5, respectively. Alignment of the capsid amino acid sequences of all FeAstV strains identified in the present study and other reference strains showed that FeAstV capsid protein could be divided into three regions, a conserved N-terminal (amino acid 1–420), a variable central region (aa 421–719) and a conserved C-terminal (aa 720–816), similar to HAstV and CaAstV. Variability analysis based on capsid amino acid sequences indicated that the amino acid identities were more than 95% in N-terminal and C-terminal, while lower than 40% in the central region among all FeAstV strains, and the majority of sequence mutations are concentrated in the central region (Fig 2). A neighbor-joining tree based on capsid amino acid sequences of FeAstV strains and other reference AstV strains from 33 mamastrovirus species and 7 avastrovirus species we constructed clearly demonstrated that all FeAstV strains identified in this study belonged to the specie MAstV 2 (Fig 3). In addition, the phylogenetic tree showed that FeAstV strains were clustered into two different groups. Most FeAstV strains identified in this study clustered together with reference strain Bristol reported in 1998 and formed FeAstV group 1. In FeAstV group 2, two identified FeAstV strains, 17CC0308 and 17CC0311, were clustered together with other FeAstV reference strains identified in USA and Hong Kong.

We then estimated the mean amino acid genetic distances among FeAstV strains between and within group. The mean amino acid genetic distances were 0.069±0.004 and 0.101±0.007 within group 1 and group2, respectively. Between group 1 and group 2, the mean amino acid genetic distance was 0.454±0.016 (Table 3).

**Table 3:** Mean amino acid distances in full-length capsid protein gene of FeAstV strains identified in the present study and other FeAstV reference strains*.

	Group 1		Group 2				
	MH253859- MH253876	AF056197	MH253877- MH253878	KM017742	KM017743	KF499111	KF374704
**MH253859-MH253876** ^a^	0.068±0.007	0.084±0.009	0.453±0.016	0.447±0.017	0.461±0.018	0.452±0.017	0.452±0.017
**MH253877-MH253878** ^b^	0.453±0.016	0.476±0.018	0.007±0.003	0.202±0.013	0.077±0.008	0.069±0.008	0.065±0.009
**Within group**	0.069±0.004		0.101±0.007				
**Between group**	0.454±0.016						

^*^ Mean amino acid distances (mean±SEM) were estimated using p-distances model with 1,000 bootstrap replicates in MEGA 7.0 software.

^a^ FeAstV strains identified in the present study except 17CC0308 and 17CC0311.

^b^ FeAstV strains 17CC0308 and 17CC0311.

## Discussion

Our study provides for the first time molecular evidence for the circulation of FeAstV in cats in northeast China. In the present study, the overall apparent prevalence of FeAstV infection in domestic cats was 23.4% (46/197), which is much higher than those reported in other countries, including Australia (4.8%, 11/228) [16], the USA (10%, 5/50) [21] and south Korea (17.7%, 11/62) [9]. However, the positive rate of FeAstV in this study was similar to that reported in Florida in 2018 [22]. The differences in the prevalence of FeAstV might be related to the sample number, sampling time and tested method. In the previous investigations, the relationship between FeAstV infection and clinical signs in cats was not been completely clarified. We found that the positive rate of FeAstV infection in cats with diarrhea (36.2%, 38/105) was higher than that in normal cats (8.7%, 8/92) in this study, and the difference was statistically significant (*p*<0.001). These results suggest that FeAstV is commonly prevalent in northeast China, and FeAstV infection is closely related to diarrhea in domestic cats. Out of 46 FeAstV-positive samples, eight were positive for FeAstV alone and 38 were co-infection with other enteroviruses including FPV, FBoV and FeKoV. Interestingly, all mixed infections of FPV were tested in cats with diarrhea (73.7%, 28/38), but no in normal cats (Table 2). Based on the high co-infection rate with FPV, we infer that FeAstV infected domestic cats might be always mixed infection with FPV, similar to a previous study [23]. FPV has been demonstrated to be an important pathogen that can cause severe diarrhea in cats, whether FeAstV infection causes diarrhea in domestic cats is not clear. So further infection experiment is needed to confirm the real pathogenic role of FeAstV in cats.

At present, astroviruses have been identified 33 species which infected mammals, and have a broad spectrum of host species including human, cat, piglet, dog, bat, cattle, sheep, mouse, rabbit, mink, sea lion and so on [4]. A great deal of investigation for astrovirus worldwide indicates that not only members of the same astrovirus species can infect different host species, but also members of different astrovirus species can infect the same host. In a previous study about that astrovirus infected in cats, four types of astrovirus, one was identified as Mamastrovirus 2, one as bat astrovirus, one was similar to fox astrovirus and one as Avastrovirus, were identified in domestic cats [22]. Phylogenetic analysis based on partial *RdRp* gene shows that all strains identified in this study share 91.1%-97.9% nucleotide identities and 92.9%-100% amino acid identities with FeAstV reference strains, and belong to the species *Mamastrovirus 2*, suggesting that Mamastrovirus 2 is most prevalent astrovirus in domestic cats. However, genetic variation cannot be adequately explained based on *RdRp* gene of FeAstV, because of that the length of available FeAstV *RdRp* sequences obtained from GenBank is limited. Hence, sequencing of the complete ORF2 gene (capsid gene) is performed in our study.

Genetic analyses based on the complete ORF2 nucleotide and deduced amino acid sequences reveal that significant sequence variation is present in FeAstV strains identified in this study. ORF2 gene encodes astrovirus capsid protein which plays a vital role in virus structure, antigenicity, infectivity and cellular localization [3,24]. ORF2 gene is also the most variable gene in all astroviruses. Alignment of amino acid sequences of astrovirus capsid protein indicates that all astroviruses have a conserved N-terminal and a variable C-terminal in the capsid protein, and the variable C-terminal half of the capsid protein mainly determines virus antigenicity [24,25]. In our study, twenty FeAstV strains shared 72.2%-99.4% amino acid identities with each other, and were divided into two different groups in the phylogenetic tree based on capsid amino acid sequences (Fig 3). Most FeAstV strains identified in the present study belonged to FeAstV group 1, and clustered together with the representative strain (AF056197) of Mamastrovirus 2. Among these strains in group 1, two strains, 17CC0502 and 17CC0714, have one-amino-acid deletion at residue 649 of capsid protein when compared with FeAstV reference strain (AF056197). Sequence alignment between the two strains and HAstV-1 showed this deletion located at the spike of the capsid protein. Considering that spike is the dominant antigen on the astrovirus surface [24], we infer this amino-acid deletion might affect FeAstV antigenicity. Samples 17CC0308 and 17CC0311 belonged to FeAstV group 2, and were 90.0%-93.9% similar to FeAstV reference strains (KM017742, KM017743, KF499111 and KF347704) reported in the USA and Hong Kong [18,19]. Variability analyses and alignment based on the capsid amino acid sequences among all FeAstV strains detected in our study and other FeAstV reference strains indicated that FeAstV capsid protein could be divided into three regions, conserved N-terminal and C-terminal, and variable central region. This result is coincident with HAstV-1 and CaAstV described previously [26,27]. According to the domain structure of HAstV capsid protein, the central region of FeAstV capsid protein encoded spike protein in the virus surface. The significant sequences variation in the central region among FeAstVs in group 1 and group 2 with lower than 40% identities, suggests that these strains might belong to different FeAstV serotypes. To confirm this view, further isolation of different FeAstV strains and serological analyses are required in our future study.

Furthermore, the phylogenetic differences in the full-length capsid gene are an important evidence for the taxonomy of astrovirus genotype species. According to the new astrovirus classification criteria submitted by the *Astrovirus* Study Group in 2010, the classification of astrovirus genotype species is performed not only on the basis of host range but also on phylogenetic differences (mean amino acid genetic distances between and within genotypes range between 0.368–0.781, and 0–0.318) in the complete capsid amino acid sequences [1,4,13]. We also estimated the mean amino acid genetic distances among all FeAstV strains based on the complete capsid amino acid sequences (Table 3). The analytic results show that the mean genetic distance between FeAstV group 1 and group 2 was 0.454±0.016 within range 0.368–0.781, suggesting that FeAstV strains should be classified into two different genotype species. FeAstV representative strain Bristol (AF056197) in group 1 was first detected in the UK in 1998, and was classified as astrovirus GI.B in Ninth ICTV report, and was identified as *Mamastrovirus* 2 by the *Astrovirus* Study Group in 2010 [4]. While representative strain 1637F (KF39911) in FeAstV group 2 was first identified in Hong Kong in 2013 [18]. The classification of genotype species in FeAstV has not been performed due to the limited sequences deposited in GenBank. Twenty complete FeAstV capsid gene sequences were obtained and used for phylogenetic analysis in this study, and the analytic result provided credible evidence for the taxonomy of FeAstV. Based on our analytic result, FeAstV strains in group 2 should be classified as a considerable novel genotype species in the genus *Masastrovirus*.

## Conclusions

In conclusion, we provide the first molecular evidence for the circulation of FeAstV in northeast China, and relevant data for the study of the FeAstV prevalence. Our study reveals that FeAstVs should be classified into two different genotype species, and considerable genetic diversity is present in FeAstV strains circulated in northeast China. Further investigations are required to understand the epidemiology and genetic variation of FeAstV in China, which will be helpful for diagnosis and prevention of this virus.

## Supporting information

S1 Table Summary of detailed information of FeAstV-positive samples identified in the present study. (DOC)

S2 Table Pairwise nucleotide and deduced amino acid identities (%) of the complete ORF2 gene among FeAstV strains detected in this study, FeAstV reference strains and other mamastrovirus strains (MAstV 1–5) obtained from GenBanK. (DOC)
