# Supplementary material for: Molecular characterization of feline astrovirus in domestic cats from Northeast China
Source: PLoS One. 2018 Oct 9;13(10):e0205441. doi: 10.1371/journal.pone.0205441 (PMC6177177; doi:10.1371/journal.pone.0205441)
Supplement: S1 Table — (DOC) [file pone.0205441.s001.doc]

**S1Table** Summary of detailed informations of FeAstV-positive samples identified in the present study.

| no. | ID | Year | Region | Source | Clinical symptom | Co-infection | GenBank accession no. | |
| --- | --- | --- | --- | --- | --- | --- | --- | --- |
|  |  |  |  |  |  |  | For RdRp gene | For the complete ORF2 gene |
| 1 | 16SY0602 | 2016 | Shenyang | ASC | Diarrhea | FBoV |  |  |
| 2 | 16SY0705 | 2016 | Shenyang | PVC | Diarrhea | - | MH253841 | MH253861 |
| 3 | 16SY0713 | 2016 | Shenyang | ASC | Diarrhea | FPV |  |  |
| 4 | 16SY0715 | 2016 | Shenyang | PVC | Diarrhea | FPV+FBoV | MH253842 | MH253862 |
| 5 | 16SY0720 | 2016 | Shenyang | PVC | Diarrhea | - |  |  |
| 6 | 17SY0902 | 2017 | Shenyang | ASC | Diarrhea | FPV |  |  |
| 7 | 17SY1204 | 2017 | Shenyang | ASC | Normal | - | MH253858 | MH253876 |
| 8 | 16JZ0608 | 2016 | Jinzhou | PVC | Diarrhea | FPV |  |  |
| 9 | 16JZ0612 | 2016 | Jinzhou | PVC | Diarrhea | - | MH253840 | MH253860 |
| 10 | 16JZ0701 | 2016 | Jinzhou | ASC | Diarrhea | FPV |  |  |
| 11 | 17JL0301 | 2017 | Jilin | PVC | Normal | FBoV |  |  |
| 12 | 17JL0302 | 2017 | Jilin | PVC | Diarrhea | FPV |  |  |
| 13 | 17JL0303 | 2017 | Jilin | PVC | Diarrhea | - | MH253854 | MH253872 |
| 14 | 17JL0310 | 2017 | Jilin | ASC | Normal | - | MH253855 | MH253873 |
| 15 | 17JL0311 | 2017 | Jilin | ASC | Diarrhea | FPV+FBoV+FeKoV |  |  |
| 16 | 17JL0312 | 2017 | Jilin | ASC | Diarrhea | FPV+FBoV+FeKoV |  |  |
| 17 | 17JL0318 | 2017 | Jilin | ASC | Diarrhea | FPV+FBoV | MH253856 | MH253875 |
| 18 | 17HRB0505 | 2017 | Harbin | PVC | Diarrhea | - | MH253851 | MH253869 |
| 19 | 17HRB0509 | 2017 | Harbin | PVC | Normal | FeKoV |  |  |
| 20 | 17HRB0511 | 2017 | Harbin | PVC | Diarrhea | FBoV | MH253852 | MH253870 |
| 21 | 17HRB0904 | 2017 | Harbin | ASC | Diarrhea | FPV |  |  |
| 22 | 17HRB0905 | 2017 | Harbin | PVC | Diarrhea | - | MH253853 | MH253871 |
| 23 | 16CC0806 | 2016 | Changchun | PVC | Diarrhea | FPV |  |  |
| 24 | 16CC1101 | 2016 | Changchun | ASC | Diarrhea | FPV+FBoV |  |  |
| 25 | 16CC1104 | 2016 | Changchun | ASC | Diarrhea | FeKoV | MH153839 | MH253859 |
| 26 | 16CC1106 | 2016 | Changchun | ASC | Diarrhea | FPV+FBoV |  |  |
| 27 | 16CC1107 | 2016 | Changchun | ASC | Normal | FBoV |  |  |
| 28 | 17CC0305 | 2017 | Changchun | ASC | Normal | FeKoV |  |  |
| 29 | 17CC0308 | 2017 | Changchun | ASC | Diarrhea | FPV+FBoV+FeKoV | MH253843 | MH253877 |
| 30 | 17CC0311 | 2017 | Changchun | ASC | Diarrhea | FPV+FBoV+FeKoV | MH253844 | MH253878 |
| 31 | 17CC0313 | 2017 | Changchun | PVC | Diarrhea | FPV |  |  |
| 32 | 17CC0502 | 2017 | Changchun | PVC | Diarrhea | - | MH253845 | MH253864 |
| 33 | 17CC0701 | 2017 | Changchun | ASC | Diarrhea | FPV |  |  |
| 34 | 17CC0704 | 2017 | Changchun | ASC | Normal | FBoV | MH253846 | MH253865 |
| 35 | 17CC0705 | 2017 | Changchun | ASC | Diarrhea | FPV |  |  |
| 36 | 17CC0709 | 2017 | Changchun | ASC | Diarrhea | FPV+FBoV |  |  |
| 37 | 17CC0711 | 2017 | Changchun | ASC | Diarrhea | FPV |  |  |
| 38 | 17CC0714 | 2017 | Changchun | ASC | Diarrhea | - | MH253847 | MH253866 |
| 39 | 17CC0801 | 2017 | Changchun | ASC | Normal | - | MH253848 | MH253863 |
| 40 | 17CC0806 | 2017 | Changchun | ASC | Diarrhea | - | MH253849 | MH253867 |
| 41 | 17CC1102 | 2017 | Changchun | ASC | Diarrhea | FPV |  |  |
| 42 | 17CC1104 | 2017 | Changchun | ASC | Diarrhea | FPV |  |  |
| 43 | 17CC1105 | 2017 | Changchun | ASC | Diarrhea | FBoV |  |  |
| 44 | 17CC1106 | 2017 | Changchun | ASC | Diarrhea | - | MH253850 | MH253868 |
| 45 | 17CC1112 | 2017 | Changchun | ASC | Diarrhea | FPV+FBoV |  |  |
| 46 | 17CC1113 | 2017 | Changchun | ASC | Diarrhea | FPV+FBoV |  |  |

ASC, animal shelter centre; PVC, private veterinary clinics.

FPV, feline parvovirus; FBoV, feline bocavirus; FeKoV, feline kobuvirus.
