# Supplementary material for: Molecular characterization of feline astrovirus in domestic cats from Northeast China
Source: PLoS One. 2018 Oct 9;13(10):e0205441. doi: 10.1371/journal.pone.0205441 (PMC6177177; doi:10.1371/journal.pone.0205441)
Supplement: S2 Table — (DOC) [file pone.0205441.s002.doc]

**S2 Table Pairwise nucleotide and deduced amino acid identities (%) of the complete ORF2 gene among FeAstV strains detected in this study, FeAstV reference strains and other mamastrovirus strains (MAstV 1-5) obtained from GenBanK.**

|  |  | Nucleotide identity (%) | |  | Amino acid identity (%) | |
| --- | --- | --- | --- | --- | --- | --- |
|  |  | MH253859-  MH253876 | MH253877-  MH253878 |  | MH253859-  MH253876 | MH253877-  MH253878 |
| MAstV 2 | MH253859-  MH253876 ^a^ | 88.4-99.7 | 76.8-83.7 |  | 93.5-99.4 | 72.2-74.6 |
|  | MH253877-  MH253878 ^b^ | 76.8-83.7 | 99.3 |  | 72.2-74.6 | 99.3 |
|  | AF056197 | 87.1-89.0 | 74.9-75.2 |  | 91.7-94.0 | 71.1-71.4 |
|  | KM017742 | 76.3-79.7 | 86.1-86.4 |  | 71.5-74.3 | 90.0-90.7 |
|  | KM017743 | 76.5-79.3 | 87.6-87.9 |  | 71.9-74.4 | 92.0-92.6 |
|  | KF499111 | 76.2-80.1 | 89.0-89.0 |  | 72.1-74.7 | 93.1-93.2 |
|  | KF374704 | 76.7-80.9 | 88.4-89.0 |  | 73.7-75.1 | 93.1-93.9 |
| MAstV 1 ^c^ | | 61.9-67.7 | 59.2-62.6 |  | 58.8-65.2 | 55.4-61.4 |
| MAstV 3 ^d^ | | 58.9-60.0 | 52.7-53.1 |  | 50.3-51.3 | 51.2-51.3 |
| MAstV 4 ^e^ | | 48.0-48.7 | 47.6-47.7 |  | 38.4-39.2 | 39.9-40.0 |
| MAstV 5 ^f^ | | 45.3-47.0 | 45.2-47.8 |  | 35.4-36.9 | 36.4-37.4 |

^a^ FeAstV strains identified in the present study except 17CC0308 and 17CC0311.

^b^ FeAstV strains 17CC0308 and 17CC0311.

^c^ Human astrovirus serotypes 1-8: HAstV-1 (L23513), HAstV-2 (l13745), HAstV-3 (AF141381), HAstV-4 (Z33883), HAstV-5 (U15136), HAstV-6 (Z46658), HAstV-7 (Y08632) and HAstV-8 (AF260508).

^d^ Porcine astrovirus genotype 1: PAstV (AB037272).

^e^ California sea lion astrovirus genotype 2: CSL2 (FJ890352).

^f^ Canine astrovirus: 3/05 (FM213330), Bari/08 (HM045005) and toSH8 (HQ623147).
